# Co-Designing Ambient Assisted Living (AAL) Environments: Unravelling the Situated Context of Informal Dementia Care

**DOI:** 10.1155/2015/720483

**Published:** 2015-06-16

**Authors:** Amy S. Hwang, Khai N. Truong, Jill I. Cameron, Eva Lindqvist, Louise Nygård, Alex Mihailidis

**Affiliations:** ^1^Rehabilitation Sciences Institute and Department of Occupational Sciences & Occupational Therapy, University of Toronto, 160-500 University Avenue, Toronto, ON, Canada M5G 1V7; ^2^Department of Software and Information Systems, University of North Carolina at Charlotte, 9201 University City Boulevard, Charlotte, NC 28223, USA; ^3^Department of Neurobiology, Care Sciences and Society, Division of Occupational Therapy, Karolinska Institutet, 141 83 Huddinge, Sweden

## Abstract

Ambient assisted living (AAL) aims to help older persons “age-in-place” and manage everyday activities using intelligent and pervasive computing technology. AAL research, however, has yet to explore how AAL might support or collaborate with informal care partners (ICPs), such as relatives and friends, who play important roles in the lives and care of persons with dementia (PwDs). In a multiphase codesign process with six (6) ICPs, we envisioned how AAL could be situated to complement their care. We used our codesigned “caregiver interface” artefacts as *triggers* to facilitate envisioning of AAL support and unpack the situated, idiosyncratic context within which AAL aims to assist. Our findings suggest that AAL should be designed to support ICPs in fashioning “do-it-yourself” solutions that complement tacitly improvised care strategies and enable them to try, observe, and adapt to solutions over time. In this way, an ICP could decide which activities to entrust to AAL support, when (i.e., scheduled or spontaneous) and how a system should provide support (i.e., using personalized prompts based on care experience), and when adaptations to system support are needed (i.e., based alerting patterns and queried reports). Future longitudinal work employing participatory, design-oriented methods with care dyads is encouraged.

## 1. Introduction

As the most important contributors to dependence and institutionalization, dementia and cognitive impairment [1] profoundly impact not only persons living with impairment, but also their significant others, relatives, and friends. While public health systems strive to assist persons with dementia (PwDs) to live at home [2], Canadian home care resources continue to fall short in meeting real-world needs [3], consequently shifting care responsibilities to informal care partners (ICPs)—most commonly family members [4]. The role of an ICP involves responding to increasing care needs and dependency over time. With or without formal support, an ICP will typically transition from supporting instrumental activities of daily living (ADLs) (e.g., finances and shopping) to assisting with basic ADLs (e.g., bathing and dressing) and to providing constant care and supervision [1]. Although the stress and burden associated with caring for a PwD is well documented (e.g., [5–9]), ICPs may wish to continue caring for as long as possible for reasons that include fulfilling filial duties [10] or continuing their relationships with PwDs [11, 12]. Taken together, there is a need for policies, services, and interventions that can better support and collaborate with ICPs in the care of PwDs [3, 13].

Concurrently, the emerging field of ambient assisted living (AAL) has positioned itself to enable older adults, including PwDs, to “age-in-place” (i.e., at home and in their communities) through the support of intelligent and pervasive computing (also referred to as “smart home”) technologies. This class of technologies aims to deliver unobtrusive, context-aware assistance by sensing and learning patterns of behaviour and, in turn, tailoring its support to specific users (e.g., [14]). Beyond studies that have aimed to demonstrate technological efficacy to this end, user studies have involved PwDs to investigate AAL applications to promote memory, safety, and functional independence in the home [15]. Although many have suggested the importance of also considering ICPs in AAL research and development [15–18], the field has yet to address how these technologies might coexist with ICPs in the care of PwDs, as opposed to replacing the care they provide. In a qualitative study with ICPs, which followed on earlier longitudinal work together with PwDs [19], Rosenberg et al. [20] found that ICPs showed overall readiness to use everyday technology to support their caring roles. In another study using home visits and interviews with PwDs and their ICPs, Wherton and Monk [21] identified dressing, medications, personal hygiene, food preparation, and social communication as potential areas for prompting and sensing technologies. Another quantitative study with ICPs concluded that these stakeholders lacked knowledge of the capabilities of intelligent technologies and recommended future user-centred design approaches to address this knowledge gap in the research process [22, 23]. This previous work recognizes ICPs as an important stakeholder group in AAL research whose needs should be considered in the design of holistic AAL solutions to meet the needs of multiple key stakeholders.

To this end, this study extends our earlier discussion of the design considerations for this context [24] to a deeper description of how ICPs envision AAL support alongside their own care of PwDs. Guided by the philosophy that AAL supporting PwDs should be designed, not to replace but rather to* complement and collaborate *with ICPs, our key study objectives were to explore (1) when or with which day-to-day, home-based activities ICPs envisioned AAL could support their care and (2) how ICPs envision interacting with the technology to specify and obtain the desired support. We pursued these objectives through an inductive codesign process with ICP participants. This participatory approach aimed to scope the needs and perspectives of ICPs in an envisaged future with AAL support; educate these stakeholders on the capabilities and potential of AAL technologies; and, together, creatively explore new possibilities for AAL design.

## 2. Method

### 2.1. Study Design

As AAL represents an imagined technological future in which the roles of ICPs have yet to be explored, our study employed a codesign approach [26] that involved ICP participants in group design workshops, followed by paper prototyping sessions with individual participants in their homes. Codesign utilizes the “collective creativity of designers working together with nondesigners” and is well suited for early stages of the design process, where complex challenges and embodiments of imagined future user experiences can be explored [26]. Integral to this process was the use of “creativity triggers”—visual artefacts that explained the concept and capabilities of AAL, guided our questions, and facilitated participants' envisioning of the design space [27]. The first trigger was an animated video demonstrating an activity-assistance AAL system, “COACH” [28], which acted as a point of departure from which participants could envision, ideate, and design their interactions with similar AAL systems. Subsequent triggers were presented in the forms of user interface designs and paper prototypes to focus participants on the codesign of a “caregiver interface”—a tool to enable an ICP to set up and specify AAL support. In this way, prototypes helped to “concretize and externalize conceptual ideas” [29] and our codesign process reflected* research through design*, an approach whereby “artefacts [are] intended to be carefully crafted questions … [that] stimulate discourse around a topic” [30]. In addition to serving as triggers, the codesigned artefacts also constituted data, together with the discussion, reflection, and interpretation they facilitated. Similar to how “technology probes” aim, in part, to collect sociological data about the contextualized use of technology [31], we focused our study on what these artefacts revealed about the needs, perspectives, and particularities of ICPs in their care contexts, rather than issues of user interface aesthetics, usability, and form factor.

### 2.2. Participants and Recruitment

Six participants were recruited from a community-based agency supporting PwDs and ICPs. Agency staff members facilitated recruitment through word-of-mouth promotion and recruitment flyers, referring all prospective participants to the research team. The first author conducted a telephone screen to qualify each prospective participant based on our study inclusion criteria: providing at least seven hours (i.e., approximately half the average provided to persons with mild dementia [32]) of unpaid care each week for a community-dwelling PwD (i.e., diagnosed or assumed dementia); assisting with most or all listed ADLs (i.e., bathing, toileting, hand-washing, toothbrushing, dressing, meal preparation, and taking medications); and having been providing care for at least six months.  Table 1 summarizes the six ICP participants who participated in Phase 1 and Phase 2, and the asterisks indicate the two participants who participated in Phase 3.

### 2.3. Our Codesign Process

**Figure 1:**
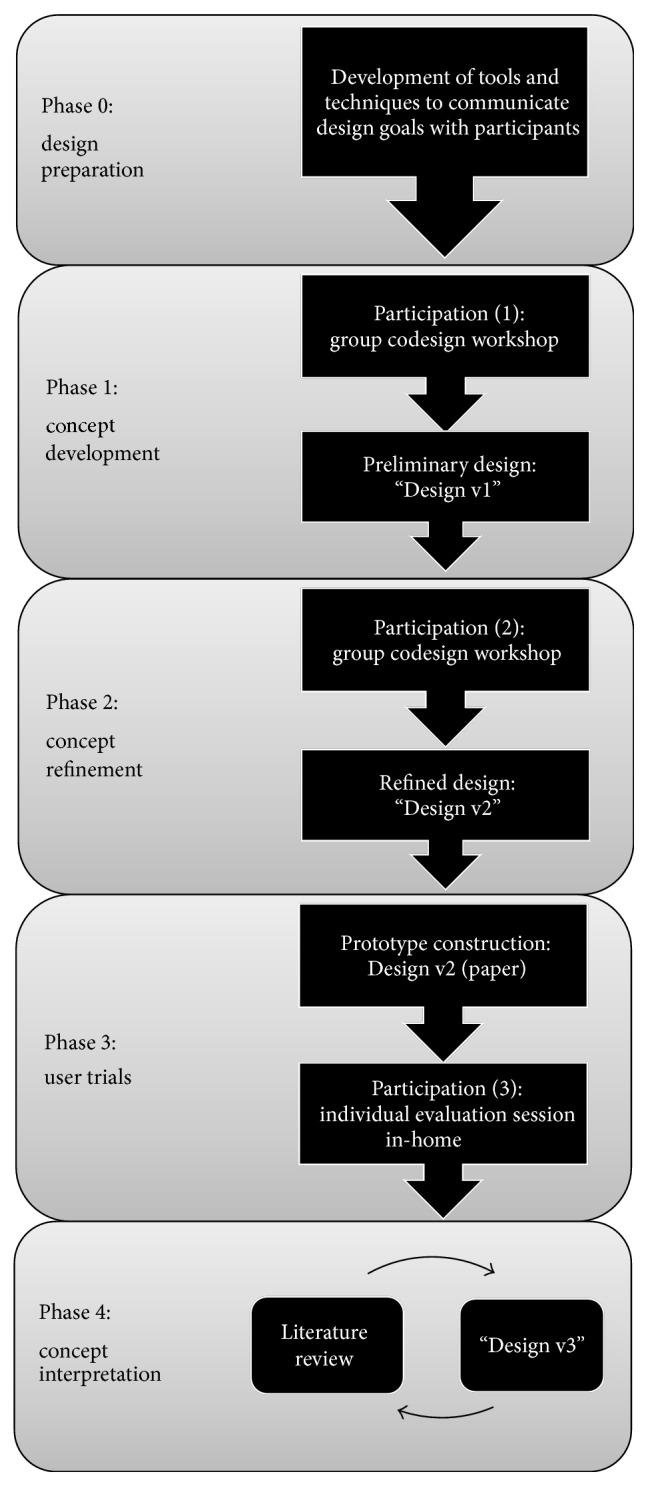
Our research/design method adapted from the USAP design model [25]. Phase 0 and Phase 4 indicate our additional/adapted stages from the original USAP model.

Our codesign method was informed by two relevant models. The conventional four-stage user-centred design (UCD) model [33]—*studying *users,* designing *for the problem space,* building *prototypes, and* evaluating *prototypes—guided our design process, and the usability, safety, attractiveness participatory (USAP) design model [25] formed the successive phases of this study, as shown in Figure 1. Moreover, our study adopted a participatory approach whereby, over multiple phases, we shared control with participants in design decisions and exchanged our respective expertise (i.e., researchers on technological capabilities and participants on informal care practices) that could then be articulated through collaboratively designed artefacts [34].

**Figure 2:**
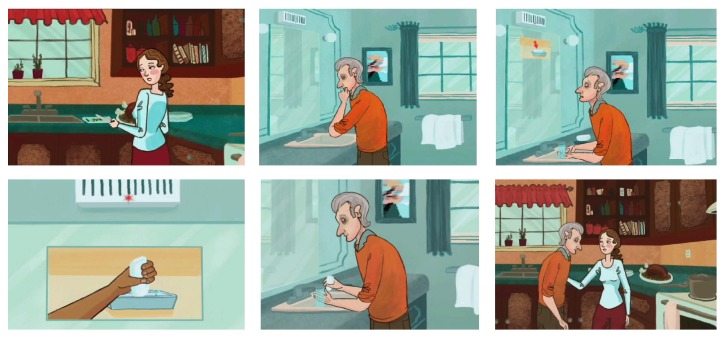
Our developed video demonstrating to participants how COACH, an AAL environment, could guide a PwD (father) in independently completing an ADL (e.g., hand-washing) while his ICP (daughter) performs other tasks.

*Phase 0: Design Preparation. *This preparatory phase aimed to set the stage for active participant engagement by developing tools to guide them in imagining a future with AAL support. We developed an animated video based on the COACH system, which has demonstrated efficacy in guiding a PwD through an ADL (e.g., hand-washing) using context-aware prompts and learning from a specific user's behaviours to improve prompting over time [28]. This video was to serve as a creativity trigger [27] to familiarize participants with the capabilities of AAL and demonstrate how an AAL system might assist a PwD who requires prompts and cues to complete an activity. As shown in Figure 2, the video depicts an older man (PwD) washing his hands in the bathroom with successive audio, picture, and video prompts from COACH, delivered only as needed, if the man experiences difficulty progressing to the next correct step of hand-washing. The man's daughter is shown in the video to be preparing dinner in the kitchen while he is able to wash his hands independently.

*Phase 1: Concept Development. *This phase aimed to address the first research objective—to explore when and with which activities ICPs envisioned AAL support. The first 90-minute group design workshop, held in the boardroom of the recruitment agency, involved a professional facilitator, the first and last authors, and the recruited participants. Upon collecting consent, we played the animated video and followed it with a discussion of participants' initial questions and comments. Participants were then given 20 minutes to complete an individual reflection/design activity: they were asked to describe (i.e., through text or sketches) how they envisioned seeking care assistance from COACH. Following this, each participant presented her idea(s) to the group, stimulating others' comments and generating new ideas. The facilitator summarized and clarified discussion themes aloud before closing the session. After the workshop, the first author reviewed field notes, participants' design submissions, audio transcripts, and workshop video. Guided by a general inductive approach [35], data were coded and categorized into activities and situations participants suggested for AAL support. Categories relevant to how participants envisioned interacting with an AAL system (i.e., our second research question) were also generated from data analysis, including aspects of AAL support participants wished to control or customize, and information they wished to receive from an AAL system. To prepare for the second workshop (Phase 2), the first author emailed a summary of findings to all participants to promote additional reflections and generated preliminary caregiver interface artefacts (“Design v1”) to trigger participants in Phase 2.

*Phase 2: Concept Refinement*. This phase aimed to steer discussion and codesign from the activities/situations for which ICPs envisioned AAL support (first research objective) toward how participants envisioned specifying and obtaining this support (second research objective). During this group design workshop, we asked participants to review, critique, annotate, and discuss their design recommendations for Design v1, first in two small groups, each of which was audiorecorded, followed by a discussion altogether. After the workshop, audio transcripts, session video, field notes, and annotated copies of Design v1 were analyzed, again using a general inductive approach [35]. This analysis generated five scenarios for specifying and obtaining AAL support: (1) setting up and orientating an AAL system for the first time; (2) modifying how the system assists the PwD with a selected activity (i.e., toothbrushing); (3) creating and customizing how the system assists the PwD with a new activity; (4) generating a report on how the PwD is responding to system assistance; and (5) using the system to “check up” on the PwD while the ICP is away from home. Following this workshop, the first author developed the next iteration of caregiver interface artefacts (“Design v2”), which would serve as triggers to participants in Phase 3.

*Phase 3: User Trials*. To continue exploring how ICPs would specify and obtain AAL support in the generated scenarios, the first author constructed a paper prototype of Design v2 and, in collaboration with the second author, developed a two-hour prototype evaluation session, guided by [36]. The session was then piloted with three domain experts affording early design recommendations, consistent cofacilitation, session timing, and anticipated responses to participants' interactions with the prototype. During the evaluation session, each of the five scenarios was posed as a task for the participant to complete. For each task, the participant read aloud the task instruction sheet (i.e., scenario description, task goals, and pertinent information needed to complete the task) before attempting to complete the task. As she progressed through the task using a pen to select actions or input information on the paper prototype, the first author flipped the prototype to the next “screen” based on her interaction. Throughout each task, the participant performed a think-aloud strategy [37] (i.e., ongoing commentary on her actions and thought processes [38]), critiqued the content and sequence of the prototype, and reflected on the appropriateness of the design to her own situation. If the participant was unsure about how to proceed in a task, the lead facilitator (second author) explained the expected actions and paused to discuss the participant's design recommendations, which were then annotated on the prototype. After all tasks were completed, we discussed the participant's overall reflections on the tasks, scenarios, prototype, and its applicability to her context. We conducted the sessions with the two selected participants in their respective homes. We selected these participants based on their interest and engagement with the research problem and process, as emphasized by [39] for participatory design in this context and participants' availability and diversity of care experiences from mild through to late-stage dementia. After both evaluation sessions, the first and second author debriefed and reviewed all field notes together with the paper prototypes annotated with participants' feedback. Over multiple discussions and the review of selected video footage (by the first author), the first and second author organized the key findings into contextual influences to specifying AAL support; AAL design “tensions”; and new design concepts for Design v3.

*Phase 4: Concept Interpretation*. This final phase involved a literature review, which aimed to facilitate our interpretation of the conceptual findings manifested in our prototypes [30] and explore new design possibilities (“Design v3”) that reflect the current knowledge base. The first author conducted a focused literature review across several databases (i.e., MEDLINE, EMBASE, PsycINFO, CINAHL, AgeLine, Scopus, Web of Science, Social Work Abstracts and ASSIA), combining the search terms (carer ^*^ OR caregiv ^*^), (burden OR strain OR stress), and (elder ^*^ OR senior ^*^ OR older adult ^*^). After reviewing titles and abstracts for relevance to ICPs of PwDs, we selected qualitative studies that discussed ICPs' care experiences, routines, and strategies of ICPs. We then synthesized and linked relevant themes to the key findings from Phase 3 (i.e., contextual influences, AAL design tensions, and new design concepts). Concurrent with the literature review, the first author collaborated with undergraduate engineering design students to produce Design v3 artefacts, which aimed to harmonize Phase 3 findings and themes from the synthesized literature.

The original study protocol and all amendments proposed throughout this multiphase study were approved by the University of Toronto Research Ethics Board (Protocol ID 26622).

## 3. Results

In the following sections, we describe our key findings across successive research phases. To address our first research objective, we first discuss the activities and situations for which participants envisioned AAL support alongside their own care. We then address our second research objective by describing how participants envisioned specifying and obtaining the desired support—by setting up and orientating the AAL system to persons and the home setting; by specifying and personalizing how the system would assist their relatives (PwDs) in activities; by scheduling and spontaneously requesting system support; and by retrieving from the system care-related information and using the system to “check up” on PwDs if and when left home unattended. Although our codesigned artefacts may refer to “COACH,” we note that this system name was maintained in discussion with participants for consistency throughout the codesign process. From the perspective of the research team, the animated video of COACH was used as trigger to help participants conceptualize the capabilities of AAL support. We contend that our findings are not limited to the capabilities of the actual COACH system but are also relevant to the broader class of AAL technologies designed to guide PwDs through home-based activities. As such, we hereafter use “the system” to refer to these technologies. In addition, as our participants were all family members of PwDs, we use “relatives” to refer to the PwDs to whom they provide care.

### 3.1. Envisioned Activities and Situations for Which AAL Can Complement Care

Overall, participants shared varying opinions about when and with which activities they would entrust or desire AAL support in the care of their relatives. They were amenable to the idea of AAL enabling their relatives to complete ADLs and other home-based activities independently, while concurrently relieving them of some care duties. Participants envisioned AAL support for several activities (i.e., hand-washing, toothbrushing, toileting, grooming, dressing, preparing and dispensing meals, making telephone calls, watching television, and gardening) but maintained mixed opinions about which they would feel comfortable entrusting to technology. Participants also enthusiastically agreed they would find it valuable to be able to create and specify how AAL could assist with “custom” activities that were particularly meaningful to their relatives. For example, Melissa felt it was important to keep her father “as independence as possible” in his valued activities:* “Gardening [was my father's] passion… so, for my mom, [she couldn't show him what to do, she could only] repeat herself, like ‘the shovel's over there, don't you see it?' So something [to help him with] tool recognition - like ‘this is what a shovel looks like… it's by the recycling bin'… [using my mom's] voice recording, or mine, or somebody familiar… to [help my father stay] a little independent.”* This finding was reinforced by our review of the literature (Phase 4) emphasizing the need to support PwDs in continuing meaningful activities [40] and maintaining as much control as possible over their everyday occupations [41]. In doing so, however, participants shared feelings of stress and frustration in having to constantly repeat information, prompts, and cues and suggested that this AAL could potentially alleviate some of this repetition. Situations demanding these reminders included orientating their relatives to day and time, helping them remember and recognize others, reminding them of the scheduled outings, and double-checking their personal belongings before outings. Ultimately, participants believed that AAL could be valuable if an ICP could select the activities and situations to which they would delegate and specify its support.

### 3.2. Specifying and Obtaining the Desired AAL Support

#### 3.2.1. Orientating the AAL Environment to Persons and Spaces in the Home

**Figure 3:**
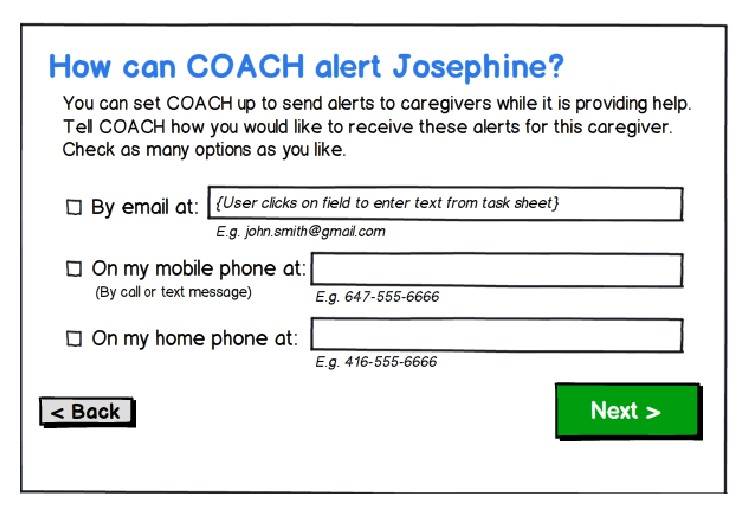
Design v2 showing how ICPs might initially set up and orientate the system to the home setting, including the protocol for sending alerts to a specific care partner.

As with familiarizing a new formal care worker to persons, care routines, and the home, participants felt that a similar orientation would be needed when specifying “personalized” support from an AAL environment, we codesigned a “setup wizard” through which an ICP could indicate which locations in the home were “augmented” with the necessary hardware (e.g., sensors, cameras) to enable AAL assistance; specify for the system who else shared in the care of their relatives (including other ICPs and formal care workers) and how they could be contacted (Figure 3); specify the preferred prompt types (e.g., verbal, picture, and video) and prompting language most suitable for their relatives; and receive an initial tutorial on how to select activities and define situational reminders for AAL support.

#### 3.2.2. Personalizing How the System Will Assist the PwD with the Selected Activities

**Figure 4:**
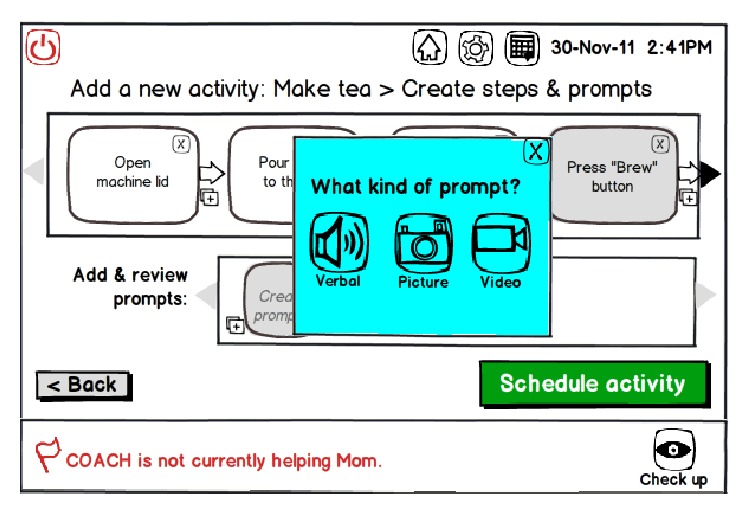
Design v2 showing how an ICP may specify an activity's steps and create (e.g., audio- or video-record) personalized prompts that they believe would assist the PwD in completing the activity (e.g., making tea).

Participants insisted that activity assistance would need to be personalized to the PwD. Over Phase 2 and Phase 3, we gradually interpreted personalization to mean specifying the appropriate prompt type, prompt content, and triggers. Participants envisioned specifying* prompt type *(e.g., verbal, picture, and video) based on their relatives' intact abilities and their own established prompting and cueing strategies. For example, some participants felt that displaying visual prompts through the mirror was clever and congruent with their own current care practices; others, however, were concerned that this may startle or confuse their relatives. To be effective,* prompt content *would need to be based on established strategies used by ICPs and be delivered in a supportive, nonpatronizing manner:* “I see how my husband reacts… if anybody would say ‘great job', he would be kind of put down… at the early stage, you really have to worry an awful lot about their pride… and consider their feelings, you can't take over.”* As it seemed the system would need to learn from and adopt ICPs' established support strategies, we codesigned caregiver interface artefacts reflecting functionality that would enable an ICP to audiorecord, take a photo/video, or select a saved media file to define a personalized prompt (Figure 4). In Phase 3, our discussions indicated the need to specify* activity steps *and* triggers *as prerequisites to defining the content of a prompt. Triggers referred to their relatives' specific actions at a particular activity step (e.g., erroneous or inappropriate action, amount of time elapsed since the PwD's last action, and verbal phrase uttered) that would signal to the system that a prompt was needed. The need for such detailed specification raised participants' concerns about the practicality of AAL, particularly in the context of caring for a relative whose fluctuating and declining abilities may demand frequent system modification. They also described prompting and cueing strategies to be highly idiosyncratic, intuitive, and tacit; explicating and “programming” this information into the system was perceived as onerous. Despite these concerns, however, we observed participants to be enthusiastic about the potential of AAL support. In particular, upon completion of Phase 3 in their respective homes, both participants engagingly recommended more naturalistic interaction methods or heuristics by which they could quickly specify AAL support for their relatives.

In Phase 4, we explored a future design concept (Design v3) that aimed to respond to this design recommendation, build on the literature in support of technology-mediated peer support between ICPs [42–47], and draw inspiration from emerging social media applications (e.g., Pinterest). The concept proposed a social network that would allow ICP “subscribers” to create, specify, and share with one another (i.e., via uploading and downloading) AAL activity support solutions using a common AAL platform. On the simplest level, sharing may enable the exchange of supportive narratives to inspire new care strategies for other ICPs. On a more sophisticated level, sharing could allow subscribers to create and upload “activity templates” containing activity's steps, prompts, and triggers, which other subscribers could then view, download, and personalize for their own use.

#### 3.2.3. Scheduling and Spontaneously Seeking AAL Support

Through codesign with participants, we also explored how ICPs could specify the appropriate timing of AAL support. In Phase 2, participants initially expressed a desire to preschedule AAL support as far in advance as possible. While completing Phase 3, however, they reflected on the practical limitations of prescheduling all system support:* “How do you program the unpredictable? How do you program something that's not routine? How do you program into the technology the specific personality of [the PwD]?”* Consistent with our review of the literature in Phase 4, improvisation was discussed as both a care strategy [48] and a natural characteristic of home life [49], which would demand sufficient flexibility for ICPs to spontaneously request, put on hold, or cancel its support as needed. Moreover, our discussion also exposed the multidimensionality of supporting home-based activities for their relatives. On a time dimension, some activities must occur at specific times (e.g., scheduled transportation pick-ups), while other activities must only be appropriately ordered (e.g., medications to be taken immediately after a meal); there are activities that are important but can occur at any time and frequency (e.g., drinking water). On a necessity dimension, there are activities that are necessary but cannot be scheduled (e.g., toileting) and others that are entirely optional (e.g., watching television). On a support dimension, certain activities might only be initiated based on their relatives' moods or abilities, an ICP's availability or stress level, or certain logistic factors. The latter led to participants' enthusiasm for AAL support in mentally or socially stimulating their relatives.

**Figure 5:**
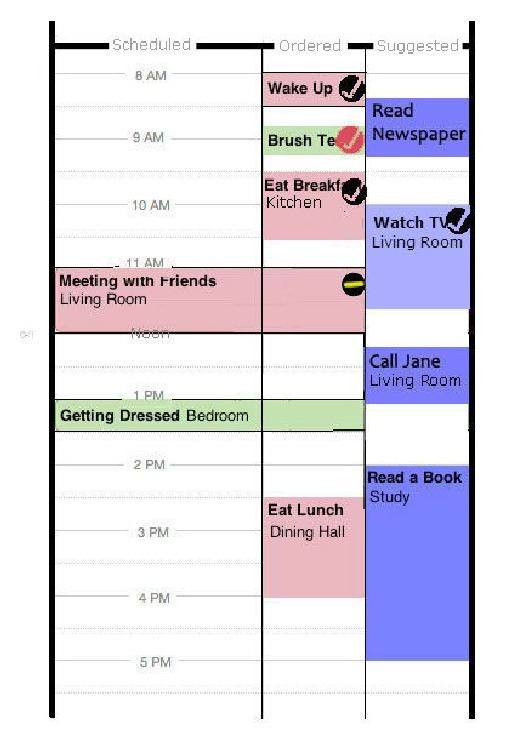
Design v3 screenshot illustrates a working mock-up of an alternative calendar design that organizes scheduled, ordered, and suggested (leisure) activities, in order of decreasing priority. The red and green coloured items indicate support by an ICP and the system, respectively. The check marks indicate whether the activity has been completed, where the red check mark indicates that an ICP was called to help the PwD complete the activity after the system's prompting was not successful. The top-right circular icon in the scheduled “Meeting with Friends” item indicates that the ICP has paused (delayed) this activity. If the scheduled item is not reinitiated after 12:15 p.m., the system may help initiate “Call Jane” if the PwD is in the living room.

In Phase 4, we translated these findings into a new design concept, reflected in Figure 5, an alternative calendar design that accommodates scheduled, ordered, and suggested (i.e., optional leisure) activities. To support improvisation, we added functionality that would allow an ICP to spontaneously cancel, pause (i.e., delay), or initiate AAL support to their relatives with leisure activities.

### 3.3. Retrieving Relevant Care Information and “Checking Up” While Away

Participants also perceived receiving care-related information from the system as another means of seeking AAL support in the care of their relatives. They agreed that being alleviated from care duties, even briefly, could be immensely valuable to them. They also shared a common desire for reassurance about if and when their relatives were home alone, though mixed opinions were discussed on which activities they would entrust to the system in their absence. Unpacking these needs and preferences over study phases led to the codesign of multiple options for conveying the desired information from the system to ICPs: “checkup” functionality, status updates, alerts, and reports.

**Figure 6:**
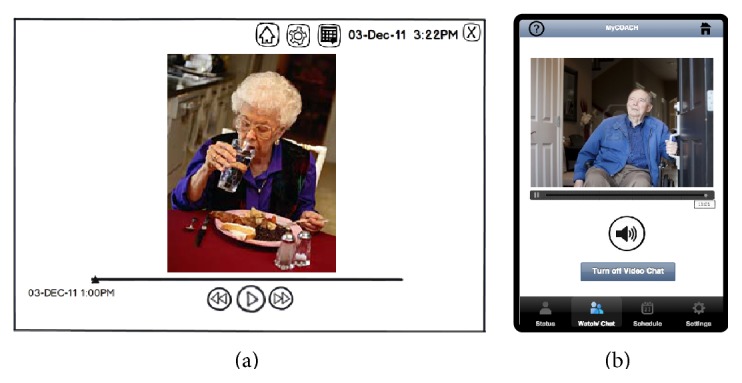
Illustrating how an ICP might be able to use the system to (a) view recorded video to check up to determine whether a PwD had completed an activity (e.g., eaten lunch) (Design v2) or (b) initiate a video call in response to an alert a potentially unsafe action that is detected (e.g., leaving the house without communicating with the ICP) (Design v3).

While being away from their relatives, participants expressed the need to “check up” if they were to entrust the system to look after their relatives in their absence. In Phase 2, we initially codesigned passive video monitoring with optional two-way video communication through the AAL system. This would allow an ICP to review video, at a later time, if activities were completed in their absence (Figure 6(a)) or check up in real time and communicate if needed. In Phase 4, however, we strived toward a more “mediating” design that could both reassure an ICP of a relative's safety while reducing “surveillance” that may only exacerbate a PwD's feeling of restricted freedom [50]. Here, we considered enabling video monitoring and communication only in situations of safety risks (e.g., wandering) (Figure 6(b)) or replacing live video with less invasive sensor data (e.g., motion, light, and temperature), as Vines et al. [51] explored in a recent telecare system field trial.

Our codesign of* status updates *also aimed to address how AAL could potentially mediate the safety versus freedom conundrum. Initially, we designed passive real-time* status updates *that were displayed on the home screen of the caregiver interface and presented in text format (e.g., “COACH is currently helping Dad brush his teeth.”). In Phase 4 (Design v3), we built on this design by adding more status details (e.g., current activity step, percentage of activity complete), speculating that this additional information may adequately reassure ICPs of their relatives' safety without the need for surveillance.

**Figure 7:**
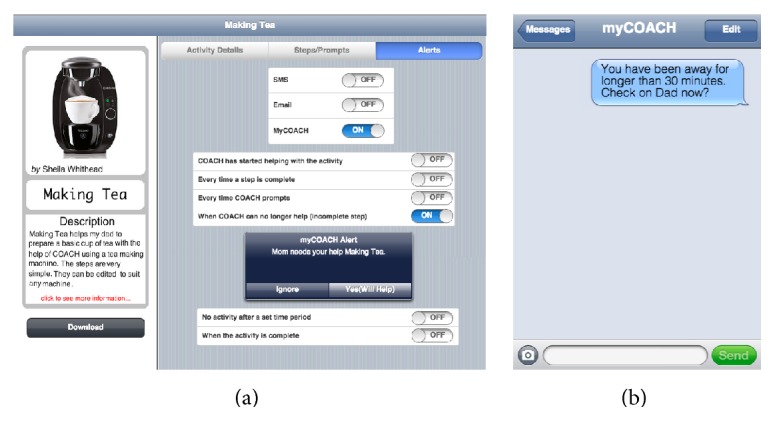
(a) Design v3 screenshot illustrating different alerting options (i.e., SMS, email, and “myCOACH” mobile application) that ICPs can specify for a particular activity and (b) sample SMS alert.

*Alerts *were another codesigned function that participants felt could afford them more peace of mind to leave their relatives at home unattended. Unsurprisingly, they wished to be immediately alerted of any potentially dangerous situations (e.g., leaving the stove on). During AAL activity assistance, if the system detected no action from a PwD over a specific time period, participants desired to be alerted for further assistance. Notably, alerts were perceived as a means of enabling a PwD to attempt activities independently while relieving ICPs of worry and constant assistance. In Phase 4, we compiled all codesigned alerting options that an ICP could specify in advance (Figure 7(a)) and explored the notion of “smart alerts,” where the system could recommend information to an ICP based on geographic location and learned patterns of information retrieval (Figure 7(b)).

**Figure 8:**
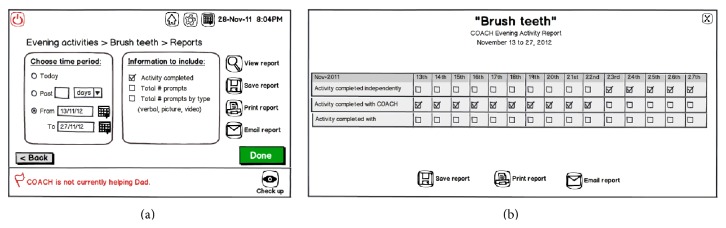
Design v2 illustrating how ICPs might (a) query and (b) retrieve reports that summarize how a PwD is managing in a particular activity with support from the system.

Lastly, participants were enthusiastic to receive from the system “on-demand” activity reports that could describe functional patterns or indicate functional decline. In Phase 3, we used Design v2, shown in Figure 8, to probe and clarify with participants their desired reporting parameters. These included: activity completion (partial or full), number of prompts (total and by type), time to activity completion, identification of problematic steps, identification of incorrect actions, and summary of alerts they received (e.g., for additional support when COACH could not longer assist). Participants anticipated that this information could signal the need for health care consultation and facilitate communication with health care providers:* “I'm not saying [there should be] printout on a regular basis, [just] as required...because sometimes my mother has a bad evening [and the] next day she's fine...but then if that runs several days in a row, you've got to know when it's time to talk to the doctor”*.

## 4. Discussion

Our findings demonstrate the need for AAL design to consider how technologies can be situated to complement the care of ICPs and emphasize the important role we expect ICPs to play in AAL customization, adoption, and ongoing use. Toward our first research objective, we learned that ICPs envisioned being able to choose which activities and situations they wished to entrust to system and indicate when they would desire this support (i.e., via care schedules or spontaneous requests). Such choices would vary based on dynamic interrelationships between home routines; their relatives' abilities, moods, and preferences; and their own availability, priorities, and emotional states. Exploring our second research objective, we gained insight into* how *ICPs envisioned specifying and obtaining AAL support. This specification may involve first-time system setup, activity selection, and detailed activity and prompt specification, processes in which ICPs would be called to translate their care expertise into system instructions. “Personalized” assistance was considered necessary for both effective support (i.e., correct activity completion) and preserving their relatives' abilities and dignity. ICPs may also desire relevant information from the system related to care. This information could be in the form of real-time monitoring and bimodal communication with their relatives, less invasive status updates on current support, alerts based on predefined triggers, and activity reports based on ICPs' specified parameters. Overall, our codesign method afforded us depth in envisioning the needs, preferences, and imagined interactions from the perspectives of ICPs. We now synthesize our findings and reflect on their strengths, limitations, and implications for future work.

Our findings reinforce that AAL technologies should be designed to be flexible, customizable, and potentially with “do-it-yourself” (DIY) capabilities to complement care routines, relationships, and experiences. From an ICP's perspective, seeking AAL support means sharing and/or turning over an aspect(s) of care, from a menial task to more complex activity assistance. Whether an ICP enlists the system to provide direct assistance (e.g., activity prompting), retrieve care-related information, and coordinate care between AAL and multiple care partners, the decision and process by which ICPs entrust care to another party cannot be taken for granted. For instance, while an ICP may find caring stressful or burdensome, he or she may also ascribe significant meaning to their care roles; they may derive a sense of pride or view caring as a natural continuation of bonds with PwDs [10, 12, 52–54]. Such mixed feelings may lend themselves to fluctuating preferences for AAL support, depending on moods, stress levels, and current circumstances. Entrusting care to a technology may also require some means of orientating and instructing the system to provide support based on ICP's established strategies. The need to explicate such detailed specifications is challenged by the often tacit, improvised nature of care routines and support strategies (e.g., prompting), which previous work confirms [48, 55]. We therefore continue to advocate (i.e., in [56, 57]) that AAL technologies should be designed with “do-it-yourself” (DIY) capabilities, to the greatest extent possible, allowing users to iteratively build and modify custom AAL solutions. First, in early-stage support, DIY capabilities may enable collaborative solution-building between ICPs and PwDs, affording both users a sense of control, whose related work stresses are a central concern for smart home users [49]. Secondly, it may allow users (i.e., again, where possible, both stakeholders) to flexibly try, modify, and scale up solutions over time, as care needs, experience, and technological proficiency evolve. As developing DIY solutions may challenge users to develop technological proficiency, doing so could promote positive feelings of mastery and self-efficacy [13], as well as reflective learning and technology adoption at one's own pace, two central principles of the “Slow Design” philosophy that aims to achieve more meaningful and sustained technology use [58, 59].

We can also extend the concept of DIY to how ICPs specify and obtain system support, problematizing this in relation to AAL technologies. Unlike most AAL approaches that “overemphasize the importance of smart devices” [17], our findings reveal that ICPs wish to maintain control in specifying, personalizing, and customizing support (e.g., activity steps, prompts, triggers, and alert preferences). Although codesign afforded us insight into their learned and largely tacit support strategies, we speculate that this assumption led to participants' concerns about the time and effort such detailed specification would demand. Ongoing work [56, 57] aims to address this by exploring more naturalistic ways in which ICPs can express and specify this information in order to iteratively build DIY AAL solutions. Moreover, to exploit the value of AAL technologies, it is also crucial to determine the appropriate degree of human interaction and control vis-à-vis the autonomy of an intelligent system—a discussion that Sun et al. [17] encourage AAL researchers to consider. Here, we may apply the Scale of Degrees of Automation [60] that places system automation and human interaction on a continuum. Applied to our context, AAL support might range from the system providing* no assistance *(i.e., the ICP assists the PwD with no AAL support); to* offering suggestions *to the ICP (i.e., AAL support with ICP's permission) and to providing fully* autonomous assistance*, where the AAL system assists without any input or confirmation from the ICP. For instance, giving an ICP the option to accept or reject AAL support in the moment may mitigate the stress of post hoc alerts from an autonomous system that is difficult to spontaneously act upon. Future work is needed to investigate the desired balance between interaction and automation in AAL applications.

Arguably, the biggest insight from this study suggests an opportunity for AAL, not only to assist a PwD while alleviating an ICP(s), but also to support both stakeholders as they transition to greater dependency. Our study provided insight into the situated context in which dependency on an ICP(s) involves learning, adapting, and negotiating with PwDs. Although our study confirmed ICPs' concerns for safety and respite [50], our participants continually advocated for the needs, values, personalities, and dignity of their relatives. The enthusiastic emphasis on enabling their relatives to continue meaningful activities was most relevant to our context and supported by studies with PwDs, even if adaptive strategies and dependency were needed [40, 41]. These findings suggest ICPs may be seeking solutions that satisfy both the needs of PwDs, for whom they advocate, and their own needs. We believe AAL solutions are positioned to play this mediating role, where ICPs and PwD can negotiate support from early stages of dependency, through a shared process of exploring and fashioning technology-enabled support strategies. In this way, this study afforded us a new conceptualization of this research/design problem, where AAL design should be based on an understanding of the contextual and temporal particularities of the “caregiving dyad” [13] and consider the “user” as the PwD together with his or her ICP(s) as an interconnected, interactional unit undergoing constant negotiation and transition.

Our described substantive findings were afforded by a fluid codesign process for which we acknowledge study limitations, strengths, and future research directions. First, our study recruited a small sample, female-only sample, from a single community-based support agency, thus, biasing the described findings to ICPs who have accessed some degree of formal care support (e.g., psychosocial, educational, and respite care) and who likely share similar cultural, socioeconomic, and environmental characteristics. Secondly, we acknowledge that participants' feedback may have been influenced or constrained by our creativity triggers, including our animated video of the COACH system, caregiver interface artefacts, and constructed scenarios/tasks. We, however, advocate for our codesign method, as it facilitated focused, productive participant involvement; richly contextualized information about current care strategies and envisioned AAL support; and enthusiastic attitudes toward AAL, as compared to previous attitudinal findings by colleagues [23]. In particular, our meticulous pilot sessions in Phase 3 allowed us to rehearse cofacilitation that would promote participants' envisioning beyond the actual capabilities of COACH or any other specific AAL system. Lastly, we recognize that this study reflects only the perspectives of these ICPs and their accounts of the needs and values of PwDs in the discussed context of AAL. As emphasized, future work should involve PwD-ICP dyads to investigate how AAL can potentially support different needs and positive relationships as dependency is negotiated over time. Our next study, for example, will involve care dyads to codesign “technology probes” [31] that can then be deployed and longitudinally studied in real-world home settings. We expect this subsequent investigation to produce a “toolkit” of design guidelines, techniques, and methods that can holistically interpret social contexts of care, creatively explore AAL design opportunities [61], and guide empathic codesign collaboration between researchers, designers, and the beneficiary end stakeholders.

## 5. Conclusion

With a better understanding of the role of AAL in everyday dementia management, we advocate that technologies should be designed to complement and collaborate with the care of ICPs to PwDs. As the care experience involves a nuanced and evolving relationship between two (or more) people, designing AAL with DIY capabilities may enable ICPs to organically craft context-appropriate solutions to support and balance the needs of PwDs with their own needs. As we attempted to reflect in this paper, delivering such capabilities relies on a situated understanding of care contexts and, most centrally, the value-driven needs of the intended technology users. To this end, we plan and encourage others toward future work that investigates PwDs together with their ICPs as an interactional user “dyad” and employs longitudinal designs with participatory, design-oriented methods to promote envisioning of experiences in a technological future.

## Figures and Tables

**Table 1:** Description of participants. The activities of daily living (ADLs) participants reported with assisting were among bathing, toileting, hand-washing, toothbrushing, dressing, meal preparation, and taking medications.

Participant (pseudonyms)	Age	Relationship	Living with the PwD?	Severity of dementia (PwD)	Assists with how many ADLs (of 7)
Jacklyn	55	Daughter	No, same apartment building	Mild to moderate	6
Heather	67	Daughter	No, within a five-minute drive	Moderate	7
Kristine	74	Spouse	Yes	Mild to moderate	7
Tabitha	77	Spouse	Yes	Moderate	7
Melissa^*^	37	Daughter	No, father was moved to nursing home two months ago	Moderate to severe	7
Hilda^*^	62	Daughter	Yes	Severe	7

The asterisks indicate the two participants selected to participate in Phase 3.

## References

[1] Prince M., Prina M., Guerchet M. (2013). *World Alzheimer Report 2013: Journey of Caring*.

[2] Alzheimer Society of Canada (2010). *Rising Tide: The Impact of Dementia on Canadian Society*.

[3] Ward-Griffin C., Hall J., Deforge R. (2012). Dementia home care resources: how are we managing?. *Journal of Aging Research*.

[4] Cranswick K., Dosman D. (2008). *Eldercare: What We Know Today*.

[5] Ory M. G., Hoffman R. R., Yee J. L., Tennstedt S., Schulz R. (1999). Prevalence and impact of caregiving: a detailed comparison between dementia and nondementia caregivers. *The Gerontologist*.

[6] Pearlin L. I., Mullan J. T., Semple S. J., Skaff M. M. (1990). Caregiving and the stress process: an overview of concepts and their measures. *Gerontologist*.

[7] Etters L., Goodall D., Harrison B. E. (2008). Caregiver burden among dementia patient caregivers: a review of the literature. *Journal of the American Academy of Nurse Practitioners*.

[8] Papastavrou E., Kalokerinou A., Papacostas S. S., Tsangari H., Sourtzi P. (2007). Caring for a relative with dementia: family caregiver burden. *Journal of Advanced Nursing*.

[9] Sörensen S., Conwell Y. (2011). Issues in dementia caregiving: effects on mental and physical health, intervention strategies, and research needs. *The American Journal of Geriatric Psychiatry*.

[10] Holroyd E. (2001). Hong Kong Chinese daughters' intergenerational caregiving obligations: a cultural model approach. *Social Science & Medicine*.

[11] Caron C. D., Bowers B. J. (2003). Deciding whether to continue, share, or relinquish caregiving: caregiver views. *Qualitative Health Research*.

[12] Quinn C., Clare L., McGuinness T., Woods R. T. (2013). Negotiating the balance: The triadic relationship between spousal caregivers, people with dementia and Admiral Nurses. *Dementia*.

[13] Nolan M., Ingram P., Watson R. (2002). Working with family carers of people with Dementia: ‘negotiated’ coping as an essential outcomeme. *Dementia*.

[14] Boger J. N., Mihailidis A. (2011). The future of intelligent assistive technologies for cognition: devices under development to support independent living and aging-with-choice. *NeuroRehabilitation*.

[15] Bharucha A. J., Anand V., Forlizzi J. (2009). Intelligent assistive technology applications to dementia care: current capabilities, limitations, and future challenges. *The American Journal of Geriatric Psychiatry*.

[16] Ding D., Cooper R. A., Pasquina P. F., Fici-Pasquina L. (2011). Sensor technology for smart homes. *Maturitas*.

[17] Sun H., Florio V. D., Gui N., Blondia C. Promises and challenges of ambient assisted living systems.

[18] Chan M., Estève D., Escriba C., Campo E. (2008). A review of smart homes—present state and future challenges. *Computer Methods and Programs in Biomedicine*.

[19] Rosenberg L., Nygard L. (2012). Persons with dementia become users of assistive technology: a study of the process. *Dementia*.

[20] Rosenberg L., Kottorp A., Nygård L. (2012). Readiness for technology use with people with dementia: the perspectives of significant others. *Journal of Applied Gerontology*.

[21] Wherton J. P., Monk A. F. (2008). Technological opportunities for supporting people with dementia who are living at home. *International Journal of Human-Computer Studies*.

[22] Czarnuch S., Hwang A. Research study debrief meeting.

[23] Czarnuch S., Mihailidis A. (2011). The design of intelligent in-home assistive technologies: assessing the needs of older adults with dementia and their caregivers. *Gerontechnology*.

[24] Hwang A. S., Truong K. N., Mihailidis A. Using participatory design to determine the needs of informal caregivers for smart home user interfaces.

[25] Demirbilek O., Demirkan H. (2004). Universal product design involving elderly users: a participatory design model. *Applied Ergonomics*.

[26] Sanders E. B. N., Westerlund B. (2011). Experiencing, exploring and experimenting in and with co-design spaces. *Proceedings of the Nordic Design Research Conference: “Making Design Matter”*.

[27] Pommeranz A., Ulgen U., Jonker C. M. Exploration of facilitation, materials and group composition in participatory design sessions.

[28] Mihailidis A., Boger J. N., Craig T., Hoey J. (2008). The COACH prompting system to assist older adults with dementia through handwashing: an efficacy study. *BMC Geriatrics*.

[29] Lim Y.-K., Stolterman E., Tenenberg J. (2008). The anatomy of prototypes: prototypes as filters, prototypes as manifestations of design ideas. *ACM Transactions on Computer-Human Interaction*.

[30] Zimmerman J., Forlizzi J., Evenson S. Research through design as a method for interaction design research in HCI.

[31] Hutchinson H., Mackay W., Westerlund B. Technology probes: inspiring design for and with families.

[32] Langa K. M., Chernew M. E., Kabeto M. U. (2001). National estimates of the quantity and cost of informal caregiving for the elderly with dementia. *Journal of General Internal Medicine*.

[33] Microsoft Research (2008). *Being Human: Human-Computer Interaction in the Year 2020*.

[34] Vines J., Clarke R., Wright P., McCarthy J., Olivier P. Configuring participation: on how we involve people in design.

[35] Thomas D. R. (2006). A general inductive approach for analysis of qualitative evaluation data. *The American Journal of Evaluation*.

[36] Snyder C. (2003). *Paper Prototyping: The Fast and Easy Way to Design and Refine User Interfaces*.

[37] Nielsen J., Clemmensen T., Yssing C. Getting access to what goes on in people's heads?: reflections on the think-aloud technique.

[38] Nielsen J., Levy J. (1994). Measuring usability: preference vs. performance. *Communications of the ACM*.

[39] Lindsay S., Brittain K., Jackson D., Ladha C., Ladha K., Olivier P. Empathy, participatory design and people with dementia.

[40] Phinney A., Chaudhury H., O'Connor D. L. (2007). Doing as much as I can do: the meaning of activity for people with dementia. *Aging and Mental Health*.

[41] Nygård L. (2004). Responses of persons with dementia to challenges in daily activities: a synthesis of findings from empirical studies. *American Journal of Occupational Therapy*.

[42] Marziali E., Damianakis T., Donahue P. (2006). Internet-based clinical services: virtual support groups for family caregivers. *Journal of Technology in Human Services*.

[43] Marziali E., Donahue P., Crossin G. (2005). Caring for others: internet health care support intervention for family caregivers of persons with Alzheimer's, stroke, or Parkinson's disease. *Families in Society*.

[44] Marziali E., Donahue P. (2006). Caring for others: internet video-conferencing group intervention for family caregivers of older adults with neurodegenerative disease. *The Gerontologist*.

[45] Chiu T., Marziali E., Colantonio A. (2009). Internet-based caregiver support for Chinese Canadians taking care of a family member with alzheimer disease and related dementia. *Canadian Journal on Aging*.

[46] Nelis S., Quinn C., Clare L. (2007). *Information and Support Interventions for Informal Caregivers of People with Dementia*.

[47] Lewis M. L., Hobday J. V., Hepburn K. W. (2010). Internet-based program for dementia caregivers. *The American Journal of Alzheimer's Disease and other Dementias*.

[48] de la Cuesta C. (2005). The craft of care: family care of relatives with advanced dementia. *Qualitative Health Research*.

[49] Davidoff S., Lee M. K., Yiu C., Zimmerman J., Dey A. K. (2006). Principles of smart home control. *UbiComp 2006: Ubiquitous Computing*.

[50] Topo P. (2009). Technology studies to meet the needs of people with dementia and their caregivers: a literature review. *Journal of Applied Gerontology*.

[51] Vines J., Lindsay S., Pritchard G. W. Making family care work: dependence, privacy and remote home monitoring telecare systems.

[52] World Health Organization (2012). *Dementia: A Public Health Priority*.

[53] Lin M. C., Macmillan M., Brown N. (2012). A grounded theory longitudinal study of carers' experiences of caring for people with dementia. *Dementia*.

[54] McDonnell E., Ryan A. A. (2014). The experience of sons caring for a parent with dementia. *Dementia*.

[55] Lindqvist E. (2012). *Assistive technology as cognitive support in everyday life for persons with dementia or stroke [Ph.D. thesis]*.

[56] Hwang A., Hoey J. Smart home, the next generation: closing the gap between users and technology.

[57] Hwang A., Hoey J. DIY smart home: narrowing the gap between users and technology.

[58] Strauss C., Fuad-Luke A. The slow design principles.

[59] Grosse-Hering B., Mason J., Aliakseyeu D., Bakker C., Desmet P. Slow design for meaningful interactions.

[60] Sheridan T. B., Parasuraman R. (2005). Human-automation interaction. *Reviews of Human Factors and Ergonomics*.

[61] Rogers Y., Scaife M., Harris E. Things aren't what they seem to be: innovation through technology inspiration.

